# Accounting for space and uncertainty in real‐time location system‐derived contact networks

**DOI:** 10.1002/ece3.6225

**Published:** 2020-04-12

**Authors:** Trevor S. Farthing, Daniel E. Dawson, Michael W. Sanderson, Cristina Lanzas

**Affiliations:** ^1^ Department of Population Health and Pathobiology College of Veterinary Medicine North Carolina State University Raleigh NC USA; ^2^ Department of Diagnostic Medicine and Pathobiology College of Veterinary Medicine Center for Outcomes Research and Epidemiology Kansas State University Manhattan KS USA

**Keywords:** animal behavior, biotelemetry, contact network, global positioning system, movement ecology, R package, radio telemetry, real‐time location

## Abstract

1. Point data obtained from real‐time location systems (RTLSs) can be processed into animal contact networks, describing instances of interaction between tracked individuals. Proximity‐based definitions of interanimal “contact,” however, may be inadequate for describing epidemiologically and sociologically relevant interactions involving body parts or other physical spaces relatively far from tracking devices. This weakness can be overcome by using polygons, rather than points, to represent tracked individuals and defining “contact” as polygon intersections.
2. We present novel procedures for deriving polygons from RTLS point data while maintaining distances and orientations associated with individuals' relocation events. We demonstrate the versatility of this methodology for network modeling using two contact network creation examples, wherein we use this procedure to create (a) interanimal physical contact networks and (b) a visual contact network. Additionally, in creating our networks, we establish another procedure to adjust definitions of “contact” to account for RTLS positional accuracy, ensuring all true contacts are likely captured and represented in our networks.
3. Using the methods described herein and the associated R package we have developed, called *contact*, researchers can derive polygons from RTLS points. Furthermore, we show that these polygons are highly versatile for contact network creation and can be used to answer a wide variety of epidemiological, ethological, and sociological research questions.
4. By introducing these methodologies and providing the means to easily apply them through the *contact* R package, we hope to vastly improve network‐model realism and researchers' ability to draw inferences from RTLS data.

## Graphical abstract

Point data obtained from real‐time location systems (RTLSs) are often processed into animal contact networks, describing instances of interaction between tracked individuals. Proximity‐based definitions of interanimal “contact,” however, are inadequate for describing epidemiologically and sociologically relevant interactions involving body parts or other physical spaces relatively far from tracking devices. To overcome this weakness, we present novel procedures for deriving polygons from RTLS point data while maintaining distances and orientations associated with individuals' relocation events, allowing researchers to define “contact” as polygon intersections.

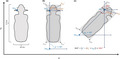

## 1 INTRODUCTION

Real‐time location systems (RTLSs) allow for spatial positioning and tracking of animate and inanimate objects in real time (Li, Chan, Wong, & Skitmore, 2016). Data sets generated by RTLSs are incredibly versatile and can be used in conjunction with other geographic data (e.g., remotely sensed data) to answer a wide variety of ecological research questions pertaining to individual‐ and population‐level animal behaviors (Kays, Crofoot, Jetz, & Wikelski, 2015). Previous studies have used RTLS data to draw inferences about (a) animals' movement speed and tortuosity (Bastille‐Rousseau et al., 2019; Liu, Xu, & Jiang, 2015; Schiffner, Fuhrmann, Reimann, & Wiltschko, 2018), (b) energy expenditures (Williams et al., 2014), (c) habitat use (Keeley, Beier, & Gagnon, 2016; Thomson et al., 2017; Tsalyuk, Kilian, Reineking, & Marcus, 2019), (d) survival and mortality rates (Klaassen et al., 2013), (e) responses to environmental stimuli (Bastille‐Rousseau et al., 2019; Tsalyuk et al., 2019), and (f) interactions with other individuals, specific locations, or environmental substrates (Chen, Sanderson, White, Amrine, & Lanzas, 2013; Chen & Lanzas, 2016; Dawson, Farthing, Sanderson, & Lanzas, 2019; Spiegel, Leu, Sih, & Bull, 2016; Theurer et al., 2012).

The most fruitful areas of RTLS data application have been disease ecology and epidemiology. Variation in contact is one of the most important drivers of disease transmission. By quantifying interanimal and environmental (i.e., relating to abiotic components of a given area) contacts, researchers can examine contact variation and the role that social behavior and spatial proximity have in shaping disease transmission in study populations (Chen et al., 2013; Dawson et al., 2019; Harris, Johnson, McDougald, & George, 2007; Leu, Kappeler, & Bull, 2011; Mersch, Crespi, & Keller, 2013; Nagy, Ákos, Biro, & Vicsek, 2010; Spiegel et al., 2016). The integration of contact data with network analysis has led to increased understanding of the drivers of contact and subsequent disease transmission (Silk, Croft, Delahay, Hodgson, Boots, et al., 2017; Silk, Croft, Delahay, Hodgson, Weber, et al., 2017). Early work by Hamede, Bashford, McCallum, and Jones (2009) showed that contact among Tasmanian devils varies between mating and nonmating season, but all members were connected in a single component, making the population highly susceptible to disease spread. Additional studies have further investigated types of social behavior and other interactions underlying transmission (Blyton, Banks, Peakall, Lindenmayer, & Gordon, 2014; Silk, Drewe, Delahay, & Weber, 2018).

Recent advances in RTLS technologies have provided researchers with the tools to more easily, accurately, and consistently identify when and for how long individuals are in contact with one another (Kays et al., 2015; Mersch et al., 2013; Pfeiffer & Stevens, 2015; Strandburg‐Peshkin, Farine, Couzin, & Crofoot, 2015). Real‐time location systems based on radio‐frequency identification (RFID) and global positioning system (GPS) technologies are becoming increasingly accurate, with positional accuracies often <2 m (Chen et al., 2013; Dawson et al., 2019; King et al., 2012; Schiffner et al., 2018), and able to fix individuals' locations over increasingly small temporal intervals (e.g., 1–10 s) (Dawson et al., 2019; Kays et al., 2015; Schiffner et al., 2018). Increases in RTLS accuracy and fix intervals translate to decreased uncertainty about animals' activities at a given time point (Kays et al., 2015; Swain, Wark, & Bishop‐Hurley, 2008). Accompanying researchers' increased ability to draw inferences about animal behavior from RTLS data, use of RTLS data in animal contact network modeling is becoming increasingly common (Krause et al., 2013; White, Forester, & Craft, 2017). In these network models, nodes (e.g., individuals and specific locations) are connected to one another by edges (i.e., contacts), which often represent instances when ≥2 nodes were observed within a specified distance threshold (SpTh) of one another (e.g., ≤1 m) over a predefined time period (Craft, 2015; Farine & Whitehead, 2015; White et al., 2017).

**FIGURE 1:**
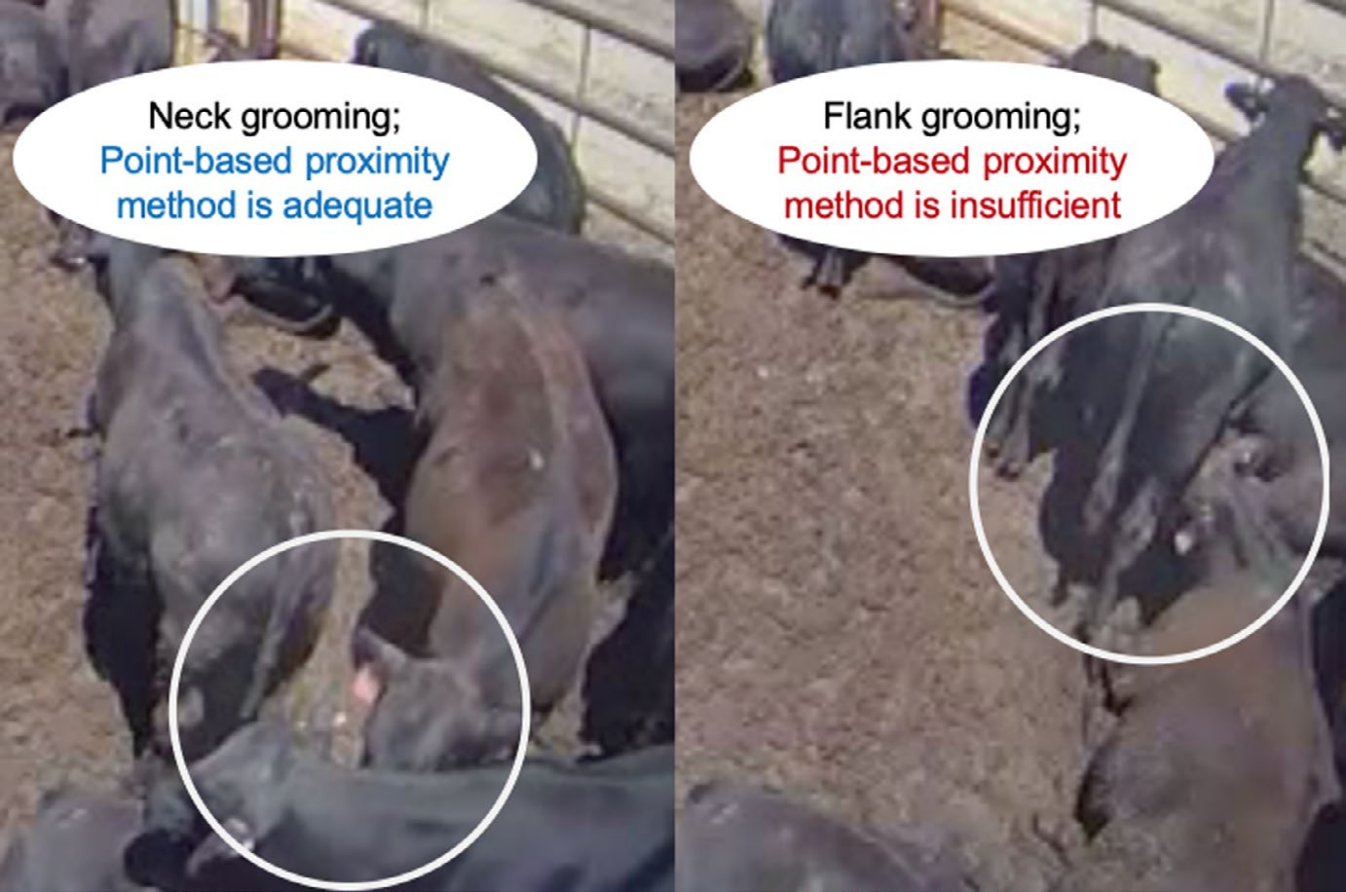
Point location‐based methods for describing tracked animal contacts may not effectively capture or characterize commonly observed interactions. Tracking devices in this example are located on animals' ears

Contact networks are frequently used to evaluate individuals' behaviors, resource use, and disease transmission risk in wildlife and livestock populations (Croft, Madden, Franks, & James, 2011; Silk, Croft, Delahay, Hodgson, Boots, et al., 2017; Silk, Croft, Delahay, Hodgson, Weber, et al., 2017), but it is often unclear if proximity‐based network edges are truly representative of real‐world pathogen transmission opportunities (Craft & Caillard, 2011; Craft, 2015; Davis, Abbasi, Shah, Telfer, & Begon, 2015). In many cases, positional accuracy is a limiting factor when deciding how to define contacts, as researchers cannot identify specific interactions between individuals (e.g., grooming and mating) if spatial accuracy is too coarse (Brookes, VanderWaal, & Ward, 2018; Leu et al., 2011). As RTLSs only produce point data, even when positional accuracy is ≈100% (i.e., approximately all RTLS‐reported coordinates correspond to individuals' true geographic locations) RTLS‐derived contact networks may represent an incomplete picture of potential contacts in a given biological system. For example, point location data collected by ear tag‐ or collar‐based tracking devices, which are often deployed in livestock‐ and wildlife‐monitoring studies, respectively (Chen et al., 2013; Dawson et al., 2019; Theurer et al., 2012; Tsalyuk et al., 2019; Strandburg‐Peshkin et al., 2015; Swain et al., 2008), are not sufficient for describing the space occupied by individuals' bodies. Therefore, contacts involving areas relatively far from the head cannot be captured without introducing substantial amounts of noise and uncertainty (Dawson et al., 2019; Figure 1).

Uncertainty related to contact precision within a relatively large SpTh leads to epidemiologically (i.e., contacts during which pathogens may be transmitted to susceptible individuals) and sociologically relevant interactions (i.e., contacts representative of specific behaviors known to indicate significant social relationships) involving body parts not equipped with tags being potentially excluded from contact networks or misidentified as noise (Blyton et al., 2014; Dawson et al., 2019). Without this information, network modelers may draw incorrect conclusions regarding the frequency of interanimal interactions (e.g., attraction or avoidance) and pathogen transmission potential in animal populations. Here, we solve this problem by describing how to incorporate animals' physical space at RTLS fix intervals into RTLS‐derived animal contact networks, ensuring that signal capture pertaining to the whole of tracked individuals' physical space is maximized. We present novel procedures for deriving polygons from RTLS point data while maintaining distances and orientations associated with individuals' relocation events (see Section 2.1) and demonstrate the versatility of this methodology for network modeling using three network creation examples (see Section 2.3). Additionally, in creating our networks, we establish a procedure to adjust definitions of “contact” to account for RTLS positional accuracy. Thus, we ensure that all true contacts in our systems of interest are likely captured and represented in generated networks. By introducing these methodologies and providing the means to easily apply them through the *contact* R package, we hope to vastly improve network‐model realism and researchers' ability to draw inferences from RTLS data.

## 2 METHODS

### 2.1 Generating polygons from RTLS data points

#### 2.1.1 Steps for polygon derivation

**FIGURE 2:**
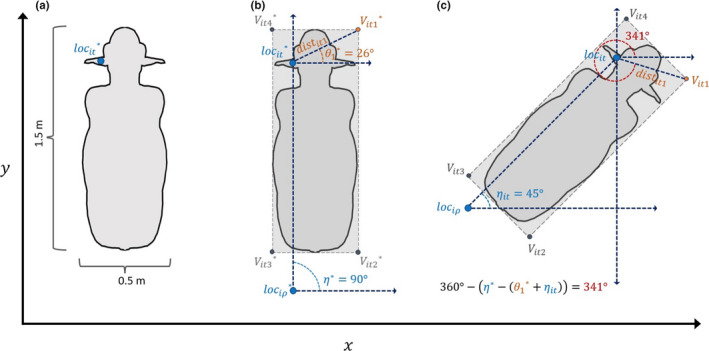
Steps for deriving polygons representing calf physical space. (a) Describe the physical dimensions of the animal and denote where the tracking device point location exists on the individual's body. (b) Describe the location of each vertex in the desired vertex set relative to tracking device location. Here, we use vertex 1 as a specific example. (c) Using the relative location information ascribed to each vertex in the planar model, interpolate empirical vertex coordinates

Accounting for objects' physical space in real time involves interpolating polygon vertices from RTLS data points. By doing so, we create 2‐dimensional objects representative of areas covered by tracked individuals' bodies from 1‐dimensional objects describing RTLS tags' point locations. Throughout this section, we refer to an example wherein we want to generate polygons covering each individual calf whose point locations are reported by a cattle monitoring RTLS (Figure 2). All terms described in Section 2.1 are listed in Table 1.

**TABLE 1:** Glossary of terms used in $\left\{{\text{poly}}_{i}\right\}$ derivation

Notation	Definition
$\left\{{\text{loc}}_{i}\right\}$	A set containing all (*x*, *y*)‐coordinate pairs describing real‐time locations of individual *i* observed during the study period.
${\text{loc}}_{\mathrm{it}}$	Denotes a single (*x*, *y*)‐coordinate pair (i.e., ( $x_{{\text{loc}}_{\mathrm{it}}}$ , $y_{{\text{loc}}_{\mathrm{it}}}$ )) within $\left\{{\text{loc}}_{i}\right\}$ describing the location of individual *i* at time *t*.
*i*	Identifies specific individuals whose locations are presented in a given real‐time location data set. Takes values 1 to *n*.
*t*	Identifies specific time points represented in a given real‐time location data set. Takes values 1 to *T*.
*T*	The total number of unique time points presented in a given real‐time location data set.
{*V_it_*}	A set containing the (*x*, *y*)‐coordinate pairs of vertices that define poly*_it_*. All vertices within a given {*V_it_*} are derived from a single point, loc*_it_*.
*V_itl_*	Denotes a single (*x*, *y*)‐coordinate pair (i.e., ( $x_{\mathrm{itl}}$ , $y_{\mathrm{itl}}$ )) within {*V_it_*}.
*l*	Identifies unique vertices contained in each {*V_it_*}. Takes values 1 to *L*.
*L*	An integer ≥ 3 describing the length of {*V_it_*}.
poly*_it_*	Area contained within vertices described in {*V_it_*}|*L*.
${\text{loc}}_{i\rho }$	The most‐recent previously reported location for individual *i* with a different (*x*, *y*)‐coordinate pair than loc*_it_* (i.e., ρ≤t-1 ).
$\eta_{\mathrm{it}}$	If gyroscopic data are available: the observed angle of movement reported by a gyroscopic measurement device (e.g., gyroscopic accelerometer) at time *t*. If no gyroscopic data are available: the absolute angle of line $\bar{{\text{loc}}_{i\rho }{\text{loc}}_{\mathrm{it}}}$ measured from a horizontal axis intersecting $loc_{i\rho }$ .
${\text{loc}}_{\mathrm{it}}^{*}$	An (*x*, *y*)‐coordinate pair in a planar model; indicates the location of loc*_it_* on individual *i* at time *t*.
${\text{loc}}_{i\rho }^{*}$	The planar‐model counterpart to ${\text{loc}}_{i\rho }$ ; describes an assumed location of ${\text{loc}}_{\mathrm{it}}^{*}$ at time ρ , and is used to identify the angular orientation of the modeled individual.
$\left\{V_{\mathrm{it}}^{*}\right\}$	A set containing the (*x*, *y*)‐coordinate pairs of vertices described in a planar model; indicates where vertices should exist relative to ${\text{loc}}_{\mathrm{it}}^{*}$ .
$V_{\mathrm{itl}}^{*}$	Denotes a single (*x*, *y*)‐coordinate pair (i.e., ( $x_{\mathrm{itl}}^{*}$ , $y_{\mathrm{itl}}^{*}$ )) within $\left\{V_{\mathrm{it}}^{*}\right\}$ .
$\eta^{*}$	The planar‐model counterpart to η ; describes the absolute angle of line $\bar{{\text{loc}}_{i\rho }^{*}{\text{loc}}_{\mathrm{it}}^{*}}$ measured from a horizontal axis intersecting ${\text{loc}}_{i\rho }^{*}$ .
${\text{dist}}_{\mathrm{itl}}$	The Euclidean distance between ${\text{loc}}_{\mathrm{it}}$ and $V_{\mathrm{itl}}$ .
$\theta_{l}^{*}$	The absolute angle of line $\bar{V_{\mathrm{itl}}^{*}{\text{loc}}_{\mathrm{it}}^{*}}$ measured from a horizontal axis intersecting ${\text{loc}}_{\mathrm{it}}^{*}$ .

For *n* tracked individuals, we define a set of planar RTLS data (*x*, *y*)‐coordinate pairs as $\left\{{\text{loc}}_{i}\right\}$ for individuals i=1,…,n , at sequential fix intervals t=1,…,T , where *T* is the total number of fix intervals over the course of the study period. Each polygon vertex, *V_itl_*, is derived from a single RTLS‐reported point location contained in $\left\{{\text{loc}}_{i}\right\}$ , loc*_it_* (i.e., ( $x_{{\text{loc}}_{\mathrm{it}}}$ , $y_{{\text{loc}}_{\mathrm{it}}}$ )) and denotes a specific (*x*, *y*)‐coordinate pair, (*x_itl_*, *y_itl_*). The variable, l=1,…,L , identifies unique poly*_it_* vertices. Each polygon, poly*_it_*, represents the area contained within the vertex set $\left.\left\{V_{\mathrm{it}}\right\}\right|L$ , where {*V_it_*} = [*V_it_*_1_,…,*V_itL_*], and *L* is an integer ≥ 3 describing the number of vertices in {*V_it_*}. For example, if ${\text{poly}}_{\mathrm{it}}$ is defined using four vertices, unique vertices in $\left.\left\{V_{\mathrm{it}}\right\}\right|L=4$ will be represented as *V_it_*_1_, *V_it_*_2_, *V_it_*_3_, and *V_it_*_4_, with respective (*x*, *y*)‐coordinate pairs (*x_it_*_1_, *y_it_*_1_), (*x_it_*_2_, *y_it_*_2_), (*x_it_*_3_, *y_it_*_3_), and (*x_it_*_4_, *x_it_*_4_).

Effectively, we want to transform each unique point location in a data set into a unique polygon with *L* vertices. Before we can derive {*V_it_*}, however, we must first consider where each *V_itl_* is located relative to a unique loc*_it_* on individuals' bodies. In other words, we know where tracking devices are located on animals' bodies (e.g., ear and neck), but before we can transform these point locations into polygon vertices, we must decide where these new points will exist on animals' bodies as well (e.g., nose and tail). In our calf example, tags are located on the left ear of each individual, and we assume animals' sizes and proportions were equivalent and stable over the observation period (Figure 2a). We decide a priori where {*V_it_*} will be located on planar, polygonal representations of space around of animals' bodies, which we refer to as “planar models” (Figure 2b). We use the star denotation to distinguish variables in planar models from their empirical counterparts (e.g., ${\text{loc}}_{\mathrm{it}}^{*}$ and $V_{\mathrm{itl}}^{*}$ ). Area described by each ${\text{poly}}_{\mathrm{it}}$ is restricted to the shape presented in these planar models, however, this limitation can be overcome to some extent by creating different models for each tracked individual, and/or updating planar models over time (*t*).

The steps for deriving {*V_it_*} coordinates while maintaining individuals' orientation at time *t* are as follows. (a) Create a planar model describing $\left\{V_{\mathrm{it}}^{*}\right\}$ and ${\text{loc}}_{\mathrm{it}}^{*}$ . (b) For a given polygon vertex *l*, calculate the hypotenuse length, ${\text{dist}}_{\mathrm{itl}}$ , for triangle $\Delta \left(x_{\mathrm{itl}}^{*},0\right)\left(0,y_{\mathrm{itl}}^{*}\right){\text{loc}}_{\mathrm{it}}^{*}$ (i.e., ${\text{dist}}_{\mathrm{itl}}=\sqrt{{\left|x_{\mathrm{itl}}^{*}-x_{{\text{loc}}_{\mathrm{it}}}^{*}\right|}^{2}+{\left|y_{\mathrm{itl}}^{*}-y_{{\text{loc}}_{\mathrm{it}}}^{*}\right|}^{2}}$ ). This is the Euclidian distance between ${\text{loc}}_{\mathrm{it}}^{*}$ and each $V_{\mathrm{itl}}^{*}$ , and is equivalent to the distance between loc*_it_* (i.e., RTLS‐reported point location) and *V_itl_* (i.e., desired polygon vertex). Once we know the distance between loc*_it_* and the vertex of interest, we can (c) identify (*x*, *y*)‐coordinate pairs that lie dist*_itl_* planar units (e.g., meters) from loc*_it_* in a $360^{\circ }-\left(\eta^{*}-\left(\theta_{l}^{*}+\eta_{\mathrm{it}}\right)\right)$ counter‐clockwise direction relative to a horizontal axis intersecting loc*_it_* (Figure 2c). This is the transformation. (d) Repeat steps 2 and 3 for each vertex *l*.

In the above formula, $\theta_{l}^{*}$ is the absolute angle of line $\bar{V_{\mathrm{itl}}^{*}{\text{loc}}_{\mathrm{it}}^{*}}$ measured from a horizontal axis in ${\text{loc}}_{\mathrm{it}}^{*}$ . The variable $\eta_{\mathrm{it}}$ is the observed angle of movement reported by a gyroscopic measurement device (e.g., gyroscopic accelerometer) at time *t* and allows us to account for changes in the orientation of animals' bodies attributed to movement while keeping $\theta_{l}^{*}$ fixed. Incorporating this variable into the {*V_it_*} derivation formula ensures that {poly*_i_*} appropriately represents animals' physical orientation (i.e., what direction they face at time *t*), which may change between times *t* and *t* + 1. In many cases, $\eta_{\mathrm{it}}$ may be unknown. For example, if gyroscopic and RTLS data were not collected concurrently (e.g., animals were outfitted with GPS transmitters, but not gyroscopic accelerometers), researchers would not intrinsically know animals' orientations. In these cases, $\eta_{\mathrm{it}}$ can be estimated by calculating the absolute angle of line $\bar{{\text{loc}}_{i\rho }{\text{loc}}_{\mathrm{it}}}$ measured from a horizontal axis intersecting ${\text{loc}}_{i\rho }$ , the most‐recent previously reported location for individual *i* with a different (*x*, *y*)‐coordinate pair than loc*_it_* (i.e., ρ≤t-1 ). The variable $\eta^{*}$ is the planar‐model counterpart to $\eta_{\mathrm{it}}$ and describes the shape's original orientation.

#### 2.1.2 Assumptions and limitations of polygon derivation

There are a couple limitations that researchers must take into account when using this procedure. Firstly, when deriving polygon vertices from RTLS points, researchers must justify how polygons relate to real‐world physical space by clearly explaining rationales for polygons' shapes, sizes, and behaviors. As previously noted, areas represented by polygons are rigid and restricted to shapes described in planar models. Though these shapes can be updated over time, to elevate the likelihood that polygons truly represent real‐world spatial features, our polygon derivation methodology is best used to model space with never‐changing or infrequently changing dimensions. For example, because the size and shape of a baboon's body frequently changes based on its activities (e.g., walking baboons are quadrupedal, but they often sit on their haunches when stationary), using our methodology to create polygons representative of baboons' physical bodies may produce inaccurate results. Conversely, as ungulates' body shapes and sizes are generally unchanging over short time periods, when modeling these species, we can be relatively confident that polygons generated using our methodology consistently reflect real‐world physical space. This is not to say that our methodology cannot be used to model regularly changing shapes, however. In these cases, researchers must utilize multiple planar models (i.e., one for each spatial form), determine criteria for switching between them (e.g., use one model when animals are observed moving slower than a specified speed, and another when their speed exceeds the stated limit), and accept that the added complexity of the system may increase risk of erroneous inference.

Secondly, in the absence of paired gyroscopic data, when $\eta_{\mathrm{it}}$ must be estimated, we must make four assumptions to account for directionality changes associated with animal movement while maintaining positional relationships between $\left\{V_{\mathrm{it}}\right\}$ and ${\text{loc}}_{\mathrm{it}}$ . First and foremost, (a) we assume that RTLS fix intervals are sufficiently small and allow RTLSs to capture all changes in animals' movement direction (i.e., animals do not face unknown directions in‐between fix intervals). The minimum required temporal resolution will vary based on the system being modeled. For example, if modeling an animal that is largely sessile and slow moving, we may assume that 10‐min fix intervals are sufficient for capturing movement directions. When modeling frequently moving animals, however, sub‐minute fix intervals are likely required to capture all directional changes. Additionally, (b) because we rely on observed animal movements to define $\eta_{\mathrm{it}}$ , we cannot know which direction animals are facing until the first relocation event occurs. Thus, we cannot create polygons representative of animals' physical orientations at the first time point, or any time points before relocations occur (i.e., in ${\text{poly}}_{\mathrm{it}}$ , t≥2 ). Furthermore, (c) we assume that individuals only move forward and in a straight line, as is common practice when calculating many path‐based movement metrics (e.g., angle of movement and step length; Miller, 2015). Finally, (d) when creating polygons representative of space occupied by animals' bodies, we assume that when the length of line $\bar{{\text{loc}}_{i(t-1)}{\text{loc}}_{\mathrm{it}}}$ is below a certain threshold (e.g., 0.1 m), individuals' physical locations and orientations remain unchanged. This immobility threshold allows us to discount orientation changes due to observed movements so miniscule that the majority of the modeled physical space is likely unaffected (e.g., head shaking), or movements caused by inaccurate RTLS reporting.

### 2.2 Network creation

#### 2.2.1 Data sets

In the following subsections, we generate direct contact (see Section 2.3.3) and visual contact (see Section 2.3.4) networks using two previously published RTLS‐generated data sets, which we refer to as *calves* and *baboons*. Neither of these data sets include any gyroscopic information about animals' movements. Therefore, as part of the polygon derivation procedure, we estimated $\eta_{\mathrm{it}}$ values using the previously described calculation and accepted the associated assumptions and limitations.

In a previous paper (Dawson et al., 2019), we published the *calves* data set, which contains RTLS data for *n* = 70 beef cattle (*Bos taurus*) calves confined in a feedlot pen. Calves were approximately 1.5 years old with estimated 1.5‐m nose‐to‐tail lengths and 0.5‐m shoulder widths. Data were obtained using a radio telemetry‐based RTLS, where 90% of points fell within ±0.5 m of individuals' true locations, at a temporal resolution of 5–10 s (i.e., fixes for each individual were obtained every 5–10 s) on 2 May 2016. To standardize the temporal resolution of this data set at 10 s, we smoothed individuals' movement paths (i.e., observed consecutive relocations) using the methodology we previously described in Dawson et al. (2019), and by doing so we obtained (*x*, *y*) coordinates representative of individuals' average location at each 10‐s interval in the study period.

The *baboons* data set, collected by Strandburg‐Peshkin et al. (2015) and made publicly available in the Movebank Data Repository (Crofoot, Kays, & Wikelski, 2015), contains geographic locations (i.e., longitude and latitude coordinate pairs) of *n* = 26 olive baboons (*Papio anubis*) living in a single troop of 46 individuals. Data were collected between 1 August and 14 August 2012 during daytime hours (i.e., between 03:00:00 and 14:59:59 UTC) using GPS collars, with ≈1‐s fix intervals and a reported average accuracy of 0.26 m (Strandburg‐Peshkin et al., 2015). To remove baboon capture‐ and handling‐induced influences from the data, we removed the first and last days of data in *baboons*. Additionally, we removed the first and last hours from each day in the data set (i.e., 03:00:00–03:59:59 UTC and 14:00:00–14:59:59 UTC). We did this because the number of individuals observed during each second of these hours was highly variable, an effect potentially caused by tracking devices powering on/off at different rates during these periods. Finally, we standardized the temporal resolution of our subset at 1‐s fix intervals by smoothing individuals' daily movement paths (Dawson et al., 2019). Thus, we were able to create a *baboons* subset containing 23 animals' geographic locations at 1‐s fix intervals between 04:00:00 and 13:59:59 UTC from 2 August to 13 August 2012. We used this subset for polygon derivation and subsequent network creation. As our polygon derivation methodology requires animals' locations to be expressed as planar coordinates, we transformed the data using an azimuthal equidistant projection centered on the data centroid (Barmore, 1991).

#### 2.2.2 Processing software

**FIGURE 3:**
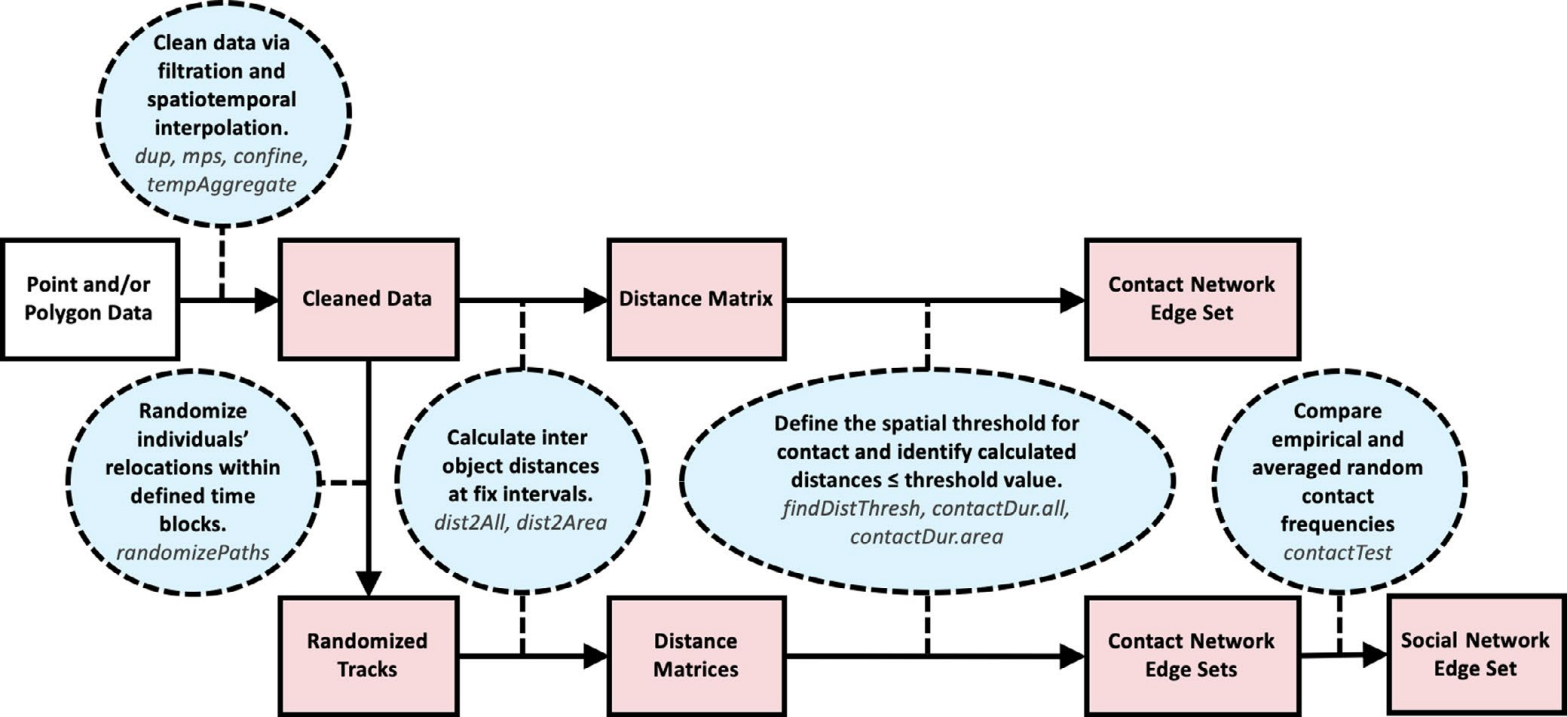
Pipeline to create time‐aggregated contact and social networks using the contact package. Blue ovals describe necessary actions and relevant package functions. Red rectangles indicate function output

To simplify polygon derivation and network creation, we developed the *contact* package for R (v. 3.6.0, R Foundation for Statistical Computing). This package is available for download on the CRAN repository and was specifically built to process spatiotemporal data into point‐ or polygon‐based contact and social networks (Figure 3). It contains 20 + functions for cleaning, interpolating, randomizing, and creating networks from spatiotemporal data, and the principal functions are briefly described in Table 2. All RTLS data processing was carried out in R using RStudio (v. 1.1.463, RStudio Team), utilized *contact* functions, and is described in Appendices S1 and S2.

**TABLE 2:** Selected contact function descriptions. Functions followed by ellipses have multiple variants referenced in text

Function	Description
*Confine*	Confinement filter; remove relocation events observed outside a specified area.
*dup*	Duplicate filter; remove duplicated relocation events.
*mps*	Meters‐per‐second filter; remove relocation events that suggest impossible/unlikely movement speeds.
*tempAggregate*	Interpolate tracked individuals' locations at specified temporal intervals.
*contactDur…*	Identify when and for how long individuals were within a specified distance threshold of one another *(contactDur.all*) or fixed locations *(contactDur.area).*
*dist2…*	Calculate planar or great‐circle distances between individual pairs *(dist2All),* or fixed locations (*dist2Area*) at every time point. Locations may be represented as points OR polygons.
*findDistThresh* ^a^	Sample from a multivariate normal distribution to create "in‐contact" point pairs based on RTLS accuracy, and generate a distribution describing average distances between point pairs.
*randomizePaths*	Generate randomized movement paths over defined temporal intervals for each individual according to methods described by Spiegel et al. (2016).
referencePoint2Polygon^a^	Generate a set of polygon vertices for each point location in a data set while maintaining individuals' angular orientation (i.e., what direction individuals are facing) at each time step.
*repositionReferencePoint* ^a^	Translates planar point locations to a different location fixed distances away, given a known angular offset, while maintaining angular orientations of movements. This function is the basis for polygon derivation from point locations, as it allows for vertex placement around planar models.
*contactTest*	Compare empirical contact distributions to null models using various testing methods (e.g., *χ* ^2^ goodness‐of‐fit, Mantel, 1967) to evaluate if observed contacts occur more or less frequently than would be expected at random, respectively.

^a^ Indicates functions based on novel procedures described within this manuscript.

#### 2.2.3 Direct contact network creation

**FIGURE 4:**
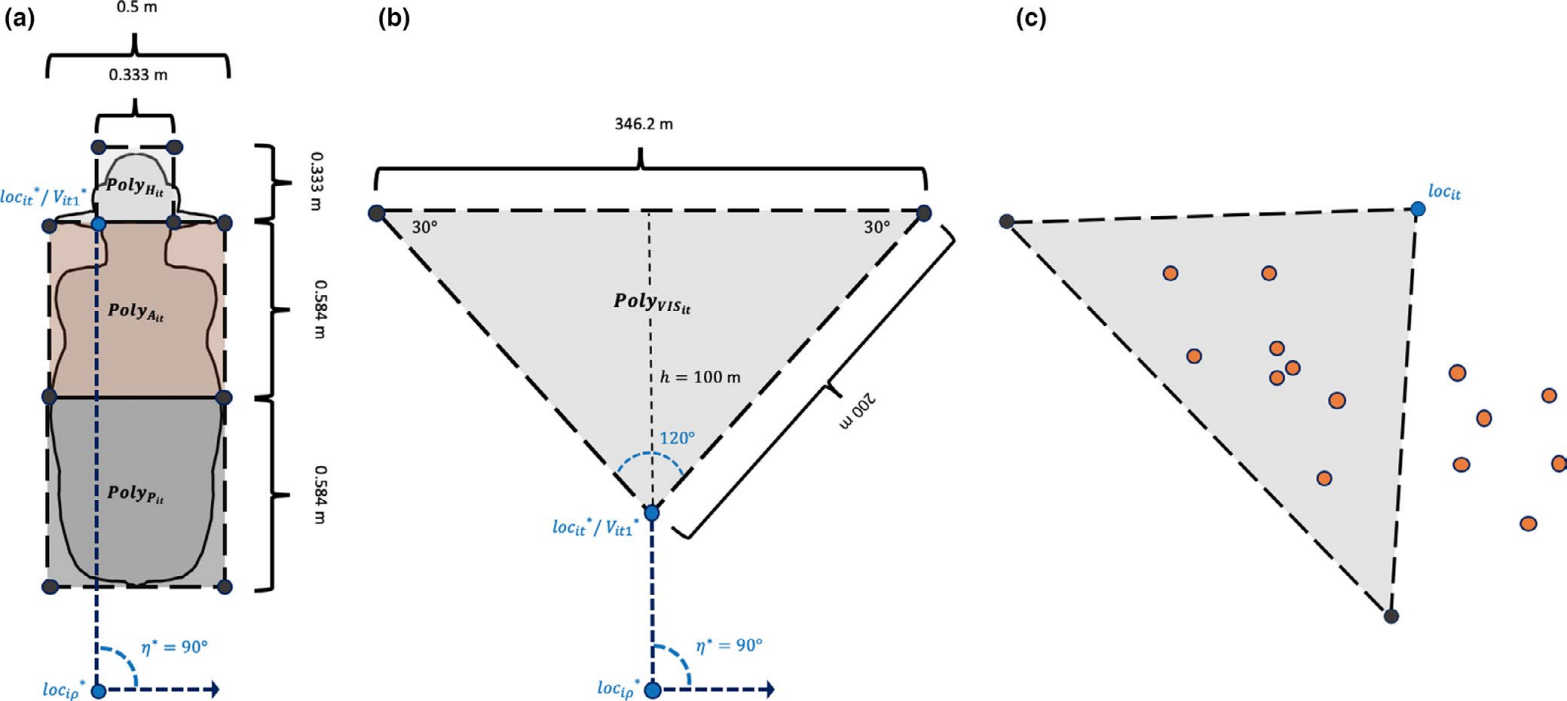
Planar models used to derive calf and baboon polygons. (a) Model of polygons representative of calves' body sections. (b) Model of polygons representing baboons' binocular visual fields up to 100 m. (c) Representation of baboon visual contacts. Orange circles represent point locations of troop members

We know that in animal populations, social interactions can increase the risk of pathogen transmission within dyads (Drewe, 2010; Blyton et al., 2014). In animal production systems, enteric pathogens (e.g., *E. coli* and *Salmonella *spp.) are often present on animals' hides, where they can be directly transmitted to hosts during social interactions or bumping (Keen & Elder, 2002; Nastasijivec, Mitrovic, & Buncic, 2013; Villarreal‐Silva et al., 2016). Because social relationships between cattle frequently involve increased physical contacts between dyad members (e.g., grooming, mounting, and butting; Gibbons, Lawrence, & Haskell, 2009; Horvath & Miller‐Coushon, 2019; MacKay, Turner, Hyslop, Deag, & Haskell, 2013), there is likely an increased risk for hide‐to‐hide or hide‐to‐mouth pathogen transfer between socially interacting individuals (Blyton et al., 2014). We aimed to create networks representative of direct contacts between calves (*i*) through which a bacterial pathogen (e.g., *E. coli*) may be transmitted from the hide of one individual to the mouth of another during the 24‐hr study period. Nodes in our contact networks are representative of physical spaces occupied by animals at any given time (*t*). Polygons delineating physical space occupancy of calves' heads (0.333 m × 0.333 m), anterior bodies (1 m × 1 m), and posterior bodies (1 m × 1 m), respectively, represented by the terms: $\left\{{\text{Poly}}_{H_{\mathrm{it}}}\right\}$ , $\left\{{\text{Poly}}_{A_{\mathrm{it}}}\right\}$ , and $\left\{{\text{Poly}}_{P_{\mathrm{it}}}\right\}$ , were derived from RFID locations and joined together to create calf polygons, $\left\{{\text{Poly}}_{{\text{Calf}}_{\mathrm{it}}}\right\}$ (i.e., ${\text{Poly}}_{{\text{Calf}}_{\mathrm{it}}}={\text{Poly}}_{H_{\mathrm{it}}}\cup {\text{Poly}}_{A_{\mathrm{it}}}\cup {\text{Poly}}_{P_{\mathrm{it}}})$ (Figure 4a). We set an immobility threshold of 0.1 m (i.e., if the data indicated individuals moved <0.1 m, their associated polygons' positions remained unchanged). We set an immobility threshold of 0.1 m (i.e., if the data indicated individuals moved <0.1 m, their associated polygons' positions remained unchanged) to account for head‐shaking events, while allowing likely true relocation events to remain unaltered.

**FIGURE 5:**
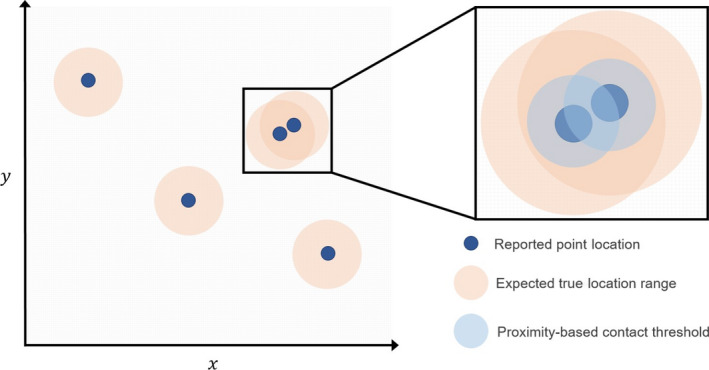
Implications for real‐time location system accuracy on proximity‐based contact determination. Reported point locations (dark blue) may not necessarily represent true locations, but rather, will fall within a certain true location range (beige). As such, in‐contact points like those shown in the inset figure may misrepresent interactions within the tracked population, if true locations actually fall outside of contact‐threshold distances (light blue) from one another

We recognize that given the positional accuracy of the *calves* data set (i.e., 90% of points within ±0.5 m of true locations), observed contacts may not be wholly representative of “true contact events” (i.e., contacts that truly happened) between individuals, as observed point locations may be erroneous (Figure 5). Assuming that RTLS errors are independent and normally distributed, we can simulate intercalf contact events by drawing x and y coordinates of hypothetical “in‐contact” point location pairs, [*x*_1_, *y*_1_, *x*_2_, *y*_2_], from a multivariate normal distribution. This distribution is parameterized such that coordinate means are [0, 0, 0, SpTh] and covariance is described by the identity function ${\left(\delta /z\right)}^{2}I$ , where δ is the radius within which RTLS points may be located around animals' true locations assuming no correlation exists between x and y coordinates (e.g., 0.5 m), and *z* is the *z*‐score associated with the probability of points falling within δ distance of true locations (e.g., for 90% of points, *z* = 1.64). Effectively, this means that given no deviation from the mean (i.e., *SD* = 0), all sampled point location pairs (i.e., [*x*_1_, *y*_1_] and [*x*_2_, *y*_2_]) will be located SpTh distance units apart from one another. Thus, paired locations in this case can be considered to be “in‐contact” with any additional distance between them resulting in cessation of contact. Introducing variation based on RTLS accuracy to multivariate sampling allows us to estimate how far apart “in‐contact” animals likely were from one another and ultimately adjust SpTh values to ensure that true contacts are likely captured and included in RTLS‐derived contact networks.

Though this is a procedure for adjusting proximity‐based contact definitions, by setting an initial SpTh value of 0, we can generate a conservative estimate of interpolygon distances required to capture true instances polygon intersections at single points. In an effort to account for the positional accuracy of the *calves* data set when defining polygonal contacts, we calculated the expected distances between 1,000,000 point location pairs with coordinates randomly sampled from a multivariate distribution, $N[\left[0,0,0,0\right],{\left(0.5/1.64\right)}^{2}I]$ . We then calculated the upper 99% CI for the resulting expected distance distribution to be used as our adjusted SpTh value for contact network creation. In this way, we estimated that a SpTh of 0.56 m likely captures ≥99% of contacts, as previously defined (i.e., polygon intersections).

**FIGURE 6:**
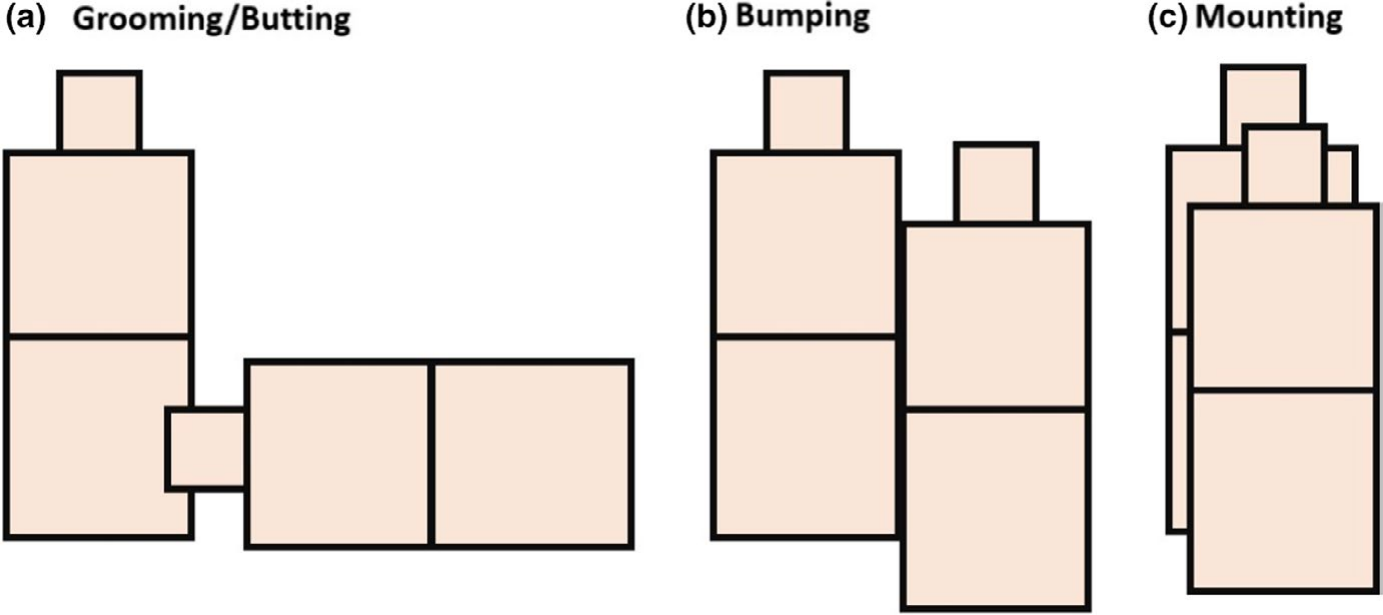
Potential interpretations of calf‐polygon intersections. (a) Head‐polygon intersections may be indicative of grooming or butting events. (b) Body‐polygon intersections may indicate intercalf bumping. (c) Concurrent intersections of multiple body sections may suggest mounting behavior. These interpretations are not wholly representative of all interaction types that may exist in the tracked population and are not mutually exclusive

**FIGURE 7:**
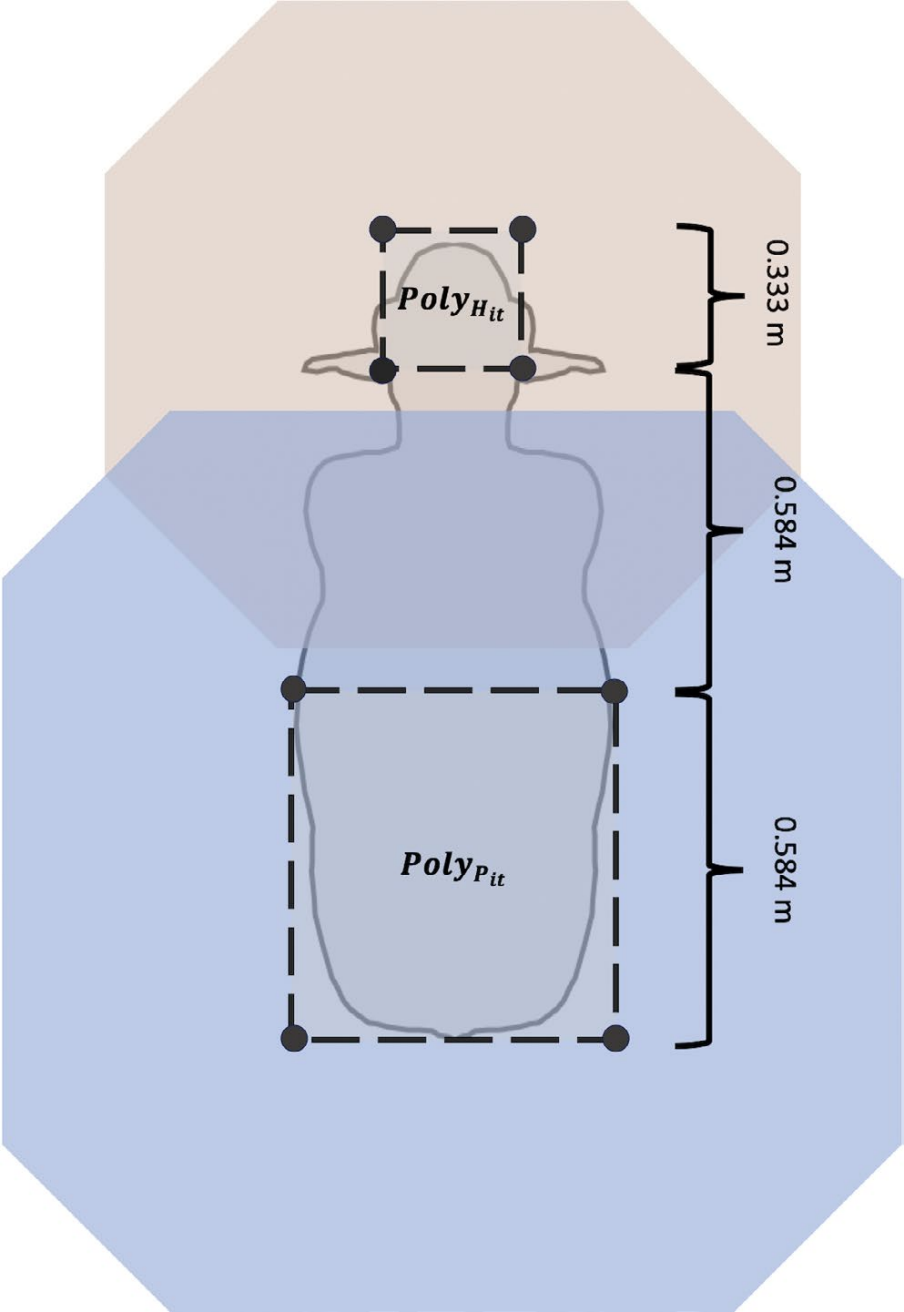
Extended 0.56‐m contact thresholds around calf‐head and calf‐posterior polygons. In “expected” contact networks, contacts occur when polygon edges are within 0.56‐m of one another

To demonstrate differences resulting from differing contact definitions, we created two distinct categories of contact network sets. In the “precise” set, contacts were said to occur when polygons intersected (i.e., SpTh = 0; Figure 6), and in the “expected” set contacts occurred when polygon edges were within 0.56 m of one another. The “expected” set can also be considered to have been created using relatively large polygons compared to the “precise” set (Figure 7). Each network set contained three time‐aggregated, undirected contact networks: (a) the “fullBody” contact network describing any instance of polygon intersection (i.e., $\sum_{i=1}^{n}\left\{{\text{Poly}}_{{\text{Calf}}_{\mathrm{it}}}\right\}\cap \left\{{\text{Poly}}_{\mathrm{Calf}}\right\}$ ) or interpolygon distances ≤ 0.56 m, (b) a “head.head” bipartite contact network describing instances when head polygons intersect (i.e., $\sum_{i=1}^{n}\left\{{\text{Poly}}_{H_{\mathrm{it}}}\right\}\cap \left\{{\text{Poly}}_{H}\right\}$ ), or are ≤0.56 m from one another, and (c) a “head.posterior” bipartite contact network describing instances when head polygons intersected (i.e., $\sum_{i=1}^{n}\left\{{\text{Poly}}_{H_{\mathrm{it}}}\right\}\cap \left\{{\text{Poly}}_{P}\right\}$ ) or were within 0.56 m of posterior polygons. In each of these networks, edge formation was limited to polygons describing different individuals (*i*) (e.g., no polygon‐based intersection can exist between ${\text{Poly}}_{H_{\mathrm{it}}}$ and ${\text{Poly}}_{A_{\mathrm{it}}}$ ). Network edges associated with each dyad were weighted by contact frequency over the 24‐hr study period. We used Welch's ANOVAs (Welch, 1947) and post hoc Games–Howell tests (Games & Howell, 1976) to evaluate differences in mean node degree, contact duration (i.e., number of consecutive time points edges existed between node pairs), and per‐capita sum contacts between all networks. Additionally, we used two‐sided Mantel tests (Mantel, 1967) to evaluate correlations between intra‐set contact matrices (i.e., “precise” and “expected” sets were evaluated separately). Mantel tests were each based on 10,000 graph permutations, and for all statistical analyses we set an α‐level of 0.05. We did not evaluate correlations between “precise” and “expected” matrices because contact definitions are mutually exclusive and would not be concurrently implemented when modeling real‐world systems. Code for polygon and network creation can be found in Appendix S1.

#### 2.2.4 Visual contact network creation

Primate social behaviors are often driven by visual cues (Bielert & Van der Walt, 1982; Janson & Di Bitetti, 1997). Recent research utilizing baboon‐tracking RTLS data has indicated that in these populations, individuals make decisions about how to move based on the movement of nearby individuals (Strandburg‐Peshkin et al., 2015), but it is unclear to what extent specific visual triggers drive these behaviors. By evaluating what behavioral cues may exist within animals' visual fields, researchers can better understand what drives decision making in study populations.

We generated a directed, time‐aggregated, bipartite visual contact network, showing when baboons were observed within the visual fields of others over the study period (Figure 4c). To do so, we first created a visual field polygon for each individual at each 1‐s timestep (*t*). This polygon set, $\left\{{\text{Poly}}_{\mathrm{VIS}}\right\}$ , was comprised of inverted triangles originating from GPS neck collar locations, with 100‐m heights, and angles of 120°, 30°, and 30° (Figure 4b). Vertex angles were based on those of human binocular visual fields (Karmakar, Pal, Majumdar, & Majumdar, 2012), as we could not identify analogous information for olive baboons. We assume all movement recorded by GPS neck collars equate to movement of associated visual field polygons. As relatively small movements may have been indicative baboon head movements potentially changing the position of their visual fields, we set an immobility threshold of 0.0 m (i.e., every observed movement, no matter how small, shifted polygons' spatial positioning).

We initially defined “contact” as occurring when a GPS point, loc*_it_*, intersected a polygon, ${\text{Poly}}_{{\text{VIS}}_{\mathrm{it}}}$ (i.e., distance between loc*_it_* and ${\text{Poly}}_{{\text{VIS}}_{\mathrm{it}}}$ equaled 0). We adjusted this SpTh to account for accuracy of the *baboons* data set (i.e., approximately 100% of points fall within ±0.26 m from true locations) using the methodology described in Section 2.3.3. By sampling 1,000,000 in‐contact point location pairs from the multivariate distribution $N[\left[0,0,0,0\right],{\left(0.26/3.89\right)}^{2}I]$ , we determined that a SpTh value of 0.109 m likely captures ≥ 99% of contacts, as previously described. Edges in this bipartite network, with independent node sets $\left\{{\text{Poly}}_{\mathrm{VIS}}\right\}$ and {loc}, were weighted by contact frequency over the study period. We report the mean per‐capita number of expected contacts per second, as well as the mean observed duration of contacts and daily node degree (i.e., number of baboons within individuals' visual fields). Code for visual contact network creation and summarization can be found in Appendix S2.

## 3 RESULTS AND DISCUSSION

### 3.1 Calf networks

All ANOVA results indicated that differences in network metrics existed, with *p*‐values < 2.2e^−16^, and post hoc Games–Howell test results are shown in Table 3. On average, “expected” contact networks consistently had greater contact durations and per‐capita sum contacts than their “precise” counterparts, highlighting the effect of relatively large SpTh values on network realization. Our results suggest that these metrics scale with polygon size. That is to say, just as increasing SpTh values lead to inflated contact frequency in point‐based proximity contact networks (Dawson et al., 2019), our work here suggests that the presence of larger polygons translates to increased probability that polygons intersect, and therefore more‐frequent and longer‐duration contact events. Average node degree generally followed the same trend, but all graphs aside from the precise‐set “head.head” one were nearly complete.

**TABLE 3:** Mean network connectivity metrics for contact networks with and without RTLS accuracy adjustment (i.e., “expected” and “precise” network sets, respectively)

Contact networks	Network density	Node degree^a^	Contact duration^a^	Per‐capita sum contacts^a^
Precise				
fullBody	1.00	68.86a (0.43)	54.28a (123.08)	3,737.23a (1,291.51)
head.head	0.88	61.06b (4.24)	7.01b (15.00)	428.11b (4.24)
head.posterior	0.99	68.42c (0.88)	27.44c (88.23)	1,877.51c (0.88)
Expected				
fullBody	1.00	68.97a (0.17)	132.40d (220.5)	9,131.97d (2,782.42)
head.head	1.00	68.80a (0.50)	40.33e (88.75)	2,774.80e (0.50)
head.posterior	1.00	68.97a (0.17)	111.30f (203.26)	7,676.43f (0.17)

^a^ Means followed by different letters differ (*p* ≤ .05) from other values within the same column according to post hoc Games–Howell tests. Standard deviations are reported in parentheses.

**FIGURE 8:**
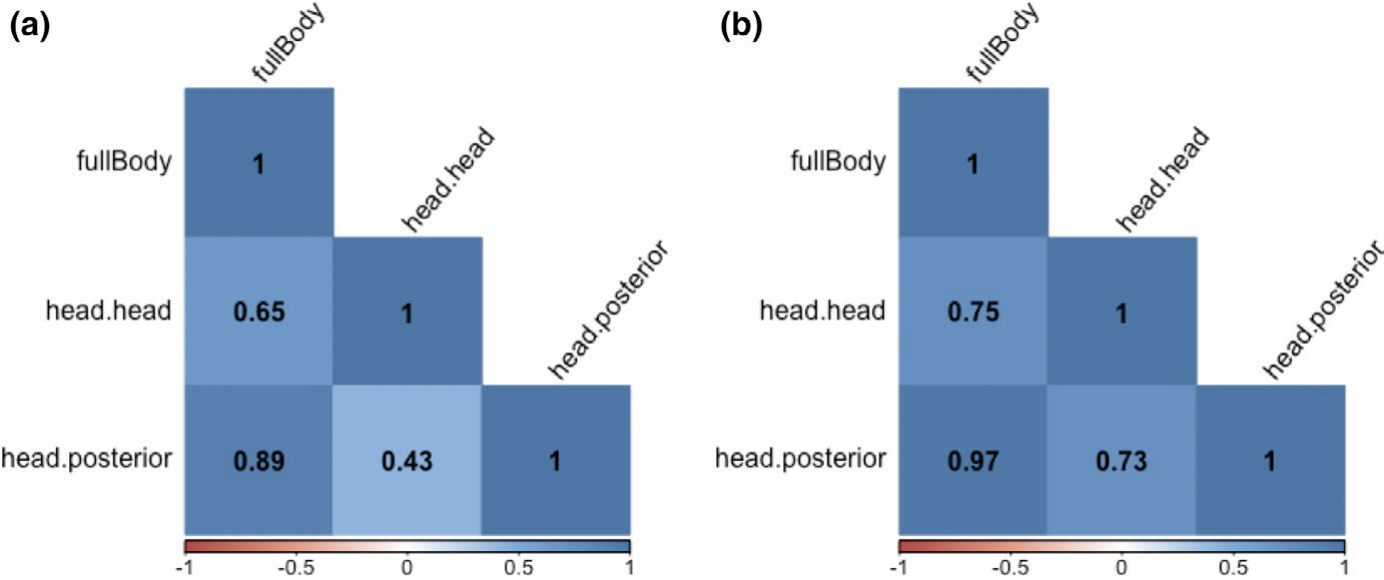
Calf network comparisons. (a) Correlation plot describing the sign and magnitude of correlations between “precise” networks. (b) Correlation plot describing the sign and magnitude of correlations between “expected” networks

We also observed strong correlations between intra‐set graphs (Figure 8). Mantel tests suggested that all intra‐set matrices were related, with a *p*‐value < .001. We found that “fullBody” graphs were consistently moderately to highly correlated with others, which is not surprising given that the “head.head” and “head.posterier” graphs were subsets of the former. Furthermore, in the case of the expected‐set “head.head” and “head.posterier” graphs, when graphs were not subsets of one another but polygons involved in contacts overlapped (Figure 7), we observed a relatively high correlation value.

The presence of a moderate correlation between the precise‐set “head.head” and “head.posterier” graphs, which did not overlap, is especially interesting. Though we did not examine specific dyadic relationships and potential correlations at the dyad level, our findings suggest that animals with more head‐to‐head contacts will likely report increased head‐to‐posterior contacts as well. This means that when modeling social relationships in cattle populations, it may be sufficient to use head‐to‐head interactions alone to identify dyads with high social affinity. On the other hand, this is not necessarily the case for modeling pathogen transmission. Assuming that our “precise” and “expected” networks reflect true interactions at least to some extent and that observed contacts are not solely a function of differences in polygon sizes, our results suggest that head‐to‐head contacts occur less frequently than head‐to‐posterior contacts, but the two contact types are inter‐related in this system. Presumably then, under the assumption that probability of transmission given contact is stable, RTLS‐derived direct pathogen transmission models of similar systems wherein only head‐to‐head contacts are effectively represented (Chen, Ilany, White, Sanderson, & Lanzas, 2015; Dawson et al., 2019) likely under‐represent dyadic interactions where pathogens may be transferred from the posterior of one animal to the mouth of the other, or vice versa.

We must note here that these findings are based on analyses of data collected over a single day and therefore may not be wholly reflective of contact patterns in this population. That said, we have demonstrated that transforming point locations into bodily polygons (e.g., animal heads, and posteriors) allows us to characterize observed contact events based on what polygons intersect (e.g., head‐to‐head). By doing so, we gain the ability to assess how different modes of contact, which may be indicative of different social behaviors (Figure 6), may affect pathogen transmission. Thus, contacts involving RTLS‐derived polygons can provide insight into both physical contact‐mediated direct pathogen transmission events, which are difficult, if not impossible, to observe in many field studies (Blyton et al., 2014).

### 3.2 Baboon network

We found that, on average, baboons observed 5.39 (*SD* = 1.02) other tagged individuals at any given second and visual contacts lasted an average of 3.67 (*SD* = 4.95) seconds. The maximum duration of a visual contact was 701 s (i.e., ≈12 min), and the average daily degree was 18.13 (*SD* = 1.74). It is necessary to note that, though we defined “visual contacts” as instances when baboon points were observed within visual field polygons, in actuality, observers may not have necessarily been actively watching “contacted” individuals during these time points (e.g., observers' eyes may have been closed, they may have been otherwise focused on other objects). Furthermore, we assumed that baboons' views were unobstructed and viewing distances were stable during the study period, which is almost certainly an oversimplification of real‐life vision. Future studies may incorporate remote‐sensing, or other geospatial data into visual field polygon generation procedures to better assess potential visual field obstruction. For example, recent work has demonstrated that LiDAR technology can be used to delineate the size and shape of individual trees in a forest (Schendryk, Broich, Tulbure, & Alexandrov, 2016). By overlaying visual field polygons onto 3D surfaces such as those described by Schendryk et al. (2016), it may be possible to introduce visual field obstruction by area vegetation in visual contact evaluation and analysis. With that in mind, however, our current results suggest baboons may closely monitor a large proportion of troop members without focusing too long on specific individuals, an act which would greatly assist with making the collective‐movement decisions described in previous work (Strandburg‐Peshkin et al., 2015).

We did not examine when resources (e.g., food and water) or observed interactions (e.g., interbaboon contacts) occurred within individuals' visual fields, but our methods can easily be used to do so. Furthermore, visual contact networks, like the one demonstrated here, can provide researchers with the means to evaluate visual cues preceding animal behaviors. By utilizing procedures for creating contact networks from RTLS data in conjunction with methodologies for analyzing animal movement patterns (Liu et al., 2015; Strandburg‐Peshkin et al., 2015; Chakravarty, Cozzi, Ozgul, & Aminian, 2019) or distance sampling procedures (Thomas et al., 2010), researchers can test hypotheses pertaining to animals' reactions to, or awareness of, visual stimuli.

### 3.3 Data processing considerations

Previous work has described in detail how difficult defining animal interactions from RTLS point data can be, as “contact” definitions must be specific to the system researchers are attempting to model (Craft, 2015; Farine & Whitehead, 2015; White et al., 2017). When defining point‐based contacts, researchers must clearly describe their rationale for selecting contact definitions, and because each definition inherently makes a number of assumptions (e.g., animals outside a given distance threshold do not pose an infection risk), network modelers must also acknowledge these unique assumptions and associated limitations in their work (Dawson et al., 2019). Defining polygon‐based contacts is less ambiguous, as interactions occur when spatial objects (i.e., points, lines, or polygons) intersect (Mersch et al., 2013). As we have demonstrated, however, much like when defining a SpTh for point location‐based contact events, researchers must take care to appropriately define the desired shape and size of desired polygons, as polygon areas likely influence downstream contact network metrics. Unfortunately, just as when defining point‐based contacts from RTLS (Dawson et al., 2019) there is no definitively “correct” polygon size and shape parameters that we can recommend. Without some kind of confirmation that contacts occurred (e.g., visual confirmation and genetic similarity), researchers must rely on assumed interactions to inform their models. In these cases, researchers must take care to ensure that their assumptions are reasonable and explicitly stated.

That said, one thing that researchers can control to some extent is the probability of capturing true contact events involving tracked individuals. The ability of RTLS data, polygon or otherwise, to describe animal contacts is ultimately constrained by RTLS accuracy. If RTLSs are 100% accurate (i.e., all reported fixes fall within ±0.0 m of true locations), researchers can be confident that observed edges in contact networks actually represent real‐world contacts. When RTLS accuracy is <100%, however, we cannot be completely sure if contacts truly occurred. In the case of the *baboons* RTLS, for example, in which points fall within ±0.26 m of true locations (Strandburg‐Peshkin et al., 2015), individuals reported to be occupying the same $loc_{\mathrm{it}}$ may actually have been up to 0.52 m apart. To account for this inherent variability, we developed the multivariate location‐sampling procedure described in Section 2.3.3. By modulating SpTh values or polygon areas for point‐ and polygon‐based contact network generation, respectively, researchers can adjust contact definitions to ensure a majority of true contact events are captured and modeled. Increasing the SpTh/polygon area using our procedure will likely introduce noise into the system (Dawson et al., 2019), but without doing so, researchers cannot be confident that a majority of real‐world contacts are truly represented in generated contact networks. Luckily, animal tracking technologies (e.g., global positioning system and radio telemetry tags) are advancing rapidly, becoming increasingly lightweight and accurate (Kays et al., 2015; Thomson et al., 2017). As these technologies advance, and newer devices are deployed, the need to inflate SpTh values will decrease, and resulting contact networks will better reflect real‐world interactions.

Aside from the aforementioned nuanced difference in how contacts are defined, polygon data can be stored and processed in much the same ways as point location data (e.g., network data can be stored as adjacency lists, and edge lists). One process that necessitates additional consideration for polygon‐based networks, however, is network randomization. Network randomization procedures traditionally involve randomizing point locations prior to contact network creation, generating null models wherein contacts occur at random, then comparing null and empirical models to test hypotheses about contact occurrence (Farine & Whitehead, 2015; Spiegel et al., 2016; Farine, 2017). Polygons derived from point locations can also be randomized to create null models using the same methodologies, but researchers must decide a priori if randomization procedures will be implemented before or after polygon generation.

If polygons are to be oriented using gyroscopic data rather than RTLS data (i.e., if researchers do not rely on observed animal movements to define $\eta_{\mathrm{it}}$ values), there would be no difference in randomization outcomes regardless of the chosen order. If polygon orientations are to be calculated using point location data alone, however, randomizing point locations prior to polygon derivation will also randomize subsequently calculated polygon orientations. Alternatively, if randomization procedures were to be implemented following polygon creation in this example (i.e., polygon locations themselves are randomized), polygon orientations will reflect those described in the empirical data set. Either randomization protocol described herein can be a useful tool for hypothesis testing and can be easily implemented through the “randomizePaths” function in our *contact* package.

## 4 CONCLUSION

Using the methods described herein and the associated *contact* package for R, researchers can derive polygons from RTLS points. We have demonstrated these polygons are highly versatile for contact network creation and can be used to answer a wide variety of epidemiological, ethological, and sociological research questions. We hope that by utilizing our methods and the tools provided, researchers can vastly improve network‐model realism and increase their abilities to draw inferences from RTLS data sets.

## CONFLICT OF INTEREST

None declared.

## AUTHOR CONTRIBUTION

**Trevor Steven Farthing:** Conceptualization (equal); Formal analysis (lead); Methodology (lead); Software (lead); Validation (lead); Visualization (lead); Writing‐original draft (lead); Writing‐review & editing (equal). **Daniel E. Dawson:** Conceptualization (equal); Software (supporting); Writing‐review & editing (equal). **Michael W. Sanderson:** Conceptualization (equal); Funding acquisition (lead); Methodology (supporting); Writing‐review & editing (equal). **Cristina Lanzas:** Conceptualization (equal); Funding acquisition (lead); Supervision (lead); Writing‐review & editing (equal). 

## Supporting information

Appendix S1

Appendix S2

## Data Availability

We utilized previously published data sets for this work. The *calves* data set (Dawson et al., 2019) can be found in the supplementary materials of our paper, “Transmission on Empirical Dynamic Contact Networks is Influenced by Data Processing Decisions” (https://doi.org/10.1016/j.epidem.2018.08.003). The *baboons* data set (Strandburg‐Peshkin et al., 2015) is available in the Movebank Data Repository (Crofoot et al., 2015—https://doi.org/10.5441/001/1.kn0816jn).
