# CORRIGENDUM

**DOI:** 10.1002/ece3.6891

**Published:** 2020-12-03

In our paper, “Accounting for space and uncertainty in real‐time location system‐derived contact networks” (*Ecol & Evol* 10(11):4702–4715), we presented a procedure for updating spatial threshold definitions to identify proximity‐based contacts from real‐time location data while accounting for real‐time location system accuracy. This is a simulation‐based procedure whereby researchers generate a distribution of *n* inter‐point distances between in‐contact point pairs, and choose an appropriate contact distance threshold that ensures a desired percentage of true positives are represented in generated contact networks. In our paper, we incorrectly state at the end of paragraph 3 in Section 2.2.3 that a spatial threshold based on the upper 99% CI for an expected‐distance distribution “likely captures ≥99% of contacts, as previously defined.” This is an erroneous statement because the CI describes the probability that the mean of the expected‐distance distribution falls within CI bounds. Thus, the statement should read that a spatial threshold based on the upper 99% CI for an expected‐distance distribution “likely captures the majority of true contacts, as previously described.” If researchers want to capture ≥99% of true contacts, any updated spatial threshold should take the value of the distribution maximum, or the mean + 3 standard deviations. Researchers should note, however, that maximizing the true positive rate for contact identification may greatly inflate the false positive rate as well.
